# Associations between Household Latrines and the Prevalence of Diarrhea in Idiofa, Democratic Republic of the Congo: A Cross-Sectional Study

**DOI:** 10.4269/ajtmh.16-0361

**Published:** 2017-06-12

**Authors:** Seungman Cha, Jae Eun Lee, Dong Sik Seo, Byoung Mann Park, Paul Mansiangi, Jae-sang Hwang, Jungwook Lee

**Affiliations:** 1 Korea International Cooperation Agency, Seongnam-si, Republic of Korea;; 2 Faculty of Infectious and Tropical Disease, Department of Disease Control, London School of Hygiene and Tropical Medicine, London, United Kingdom;; 3 Korea Environment Corporation, Incheon, Republic of Korea;; 4 Kinshasa University, Kinshasa, Democratic Republic of the Congo

## Abstract

Despite the importance of sanitation, few studies have assessed the effects of latrines on the health outcomes of children under 5 years of age. We assessed the relations between latrine coverage and the prevalence of diarrhea in children under 4 years of age. In this cross-sectional study, we analyzed the baseline data obtained as part of a longitudinal survey targeting 720 households in Idiofa, Bandundu, Democratic Republic of the Congo. We categorized latrines according to the presence of each major component and investigated whether diarrhea prevalence of children under 4 years of age is associated with latrine availability and improvement. Latrines have health benefits regardless of whether they are improved. Also worth noting is that comparatively well-equipped and more appropriately managed latrines could prevent child diarrhea more effectively than less equipped or inappropriately managed latrines. Households who have a latrine with a superstructure, roof, and no flies (a partly improved latrine) were found to be 52% less likely to report cases of diarrhea than households with unimproved latrines (adjusted odds ratio [OR] = 0.48, confidence interval [CI] = 0.31–0.76), which are all the other latrines not included in the partly improved latrine category. We have observed the profound protective effect of latrines with a superstructure. This study demonstrates that latrines are associated with significant improvements in health even when they do not fully meet the conditions of improved latrines. This study adds value to the limited evidence on the effect of latrines on health parameters by demonstrating that latrines have correlations with health benefits regardless of whether they are improved, as well as by elucidating the most essential components of improved latrines.

## INTRODUCTION

An estimated 2.4 billion people do not have access to improved sanitation, as defined by the World Health Organization/United Nations Children’s Fund (WHO/UNICEF) Joint Monitoring Program, and 946 million people openly defecate,^1^ including an estimated 7.1 million in the Democratic Republic of the Congo (DRC).^2–4^ Diarrhea accounted for 9.2% of deaths in children under 5 years of age (U5C) in the DRC in 2013, and inadequate sanitation and poor hygiene are the main factors that contribute to the disease burden of diarrhea.^5,6^

The DRC has an extremely high mortality rate for U5C (119 out of 1,000 live births in 2013), exceeded only by seven countries in which diarrheal disease is responsible for approximately 11% of child deaths.^3^ Moreover, the DRC lags behind other countries in sub-Saharan Africa in terms of water and sanitation coverage. Only 16% of households in the DRC have access to improved sanitation, including 36% of urban households and 4% of rural households, compared with an average of 44% and 24% of urban and rural households, respectively, in other countries in sub-Saharan Africa overall.^4^

Despite the importance of sanitation, there has been little sound evidence published so far on to what extent the availability and utilization of latrines can reduce diarrheal prevalence.^7–19^ Recent RCT studies did not fully demonstrate the net impact of latrines on health outcomes of U5C due to their methodological limitations or insufficient coverage with regard to improved latrine uptake and utilization. The previous studies failed to increase latrine coverage to a universal level or even a level sufficient to achieve the herd protection effect of sanitation^7^ at community level.^8–20^ To quantify the effect of increased latrine coverage on the reduction of child diarrhea, we are currently carrying out an experiment involving cluster-randomized controlled trials (cRCTs) in the DRC. A *quartier*, a subdivision of a village, is to be the randomization unit for this cluster-randomized trial, as it is likely to be a cluster in which improved sanitation can have a protective effect on diarrheal transmission. 18 *quartiers* were randomly selected as study areas for this trial and each allocated to the intervention or control arm based on the baseline survey results in January 2015. The study will use a phase-in design, in which only the intervention arm will receive the intervention for the first phase and then the intervention will roll out in the control arm. In this cross-sectional study, we analyzed the baseline data obtained in January 2015 as part of a longitudinal survey along with the trial targeting 720 households in the study area of Idiofa Territory, Bandundu Province, DRC.

We aimed to assess the relationship between latrine coverage and the prevalence of diarrhea in children, as well as to compare households with and without latrines with regard to demographic and socioeconomic characteristics and factors related to water, sanitation, and hygiene (WASH) to better understand key factors of latrine uptake. Unlike previous studies that have explored the effects of the availability of latrines,^8–20^ we examine whether latrine management as well as latrine availability are associated with health benefits. In addition to the access to sanitation facilities, it is critical to prevent the transmission of pathogens while using a latrine. In this sense, we analyze whether latrine management as well as latrine coverage can reduce the occurrence of U4C diarrhea. We also evaluated the effects of intermediate factors, such as feces around the pit hole and the presence of flies, which were not thoroughly assessed in previous studies. Constructing latrines can prevent the spread of disease as a primary barrier to preventing pathogens from transmitting via flies, fields/floors, and drinking water contamination.^21^ If latrines are badly constructed, poorly managed, or used inappropriately, they can be potential disease transmission routes.^22^ Therefore, we paid attention to the good design and appropriate management of latrines—functioning appropriately to disrupt disease transmission.

Reflecting the 5-f diagram, we initially intended to assess the association of disrupting the transmission cycle (field, flies, and fluid) with diarrheal prevalence to produce evidence for what an improved latrine should look like. In this regard, we attempted to assess the association between latrine structure (pit-hole depth, pit-hole cover, cemented slab, and handwashing facility) and intermediate factors (feces around the pit-hole and flies). However, we found no households with sufficient pit-hole depth, pit-hole cover, cemented slab, and handwashing facility at baseline in the study area. Thus, we shifted our attention to exploring the association of intermediate factors (feces around the pit-hole, flies) with child diarrheal prevalence so that we could generate insight into the effects of improved latrine.

This cross-sectional study adds important information to the body of evidence regarding the effects of latrines on health outcomes, although its design does not permit causal inference.

## METHODS

### Ethics statement.

This study was approved by the Institutional Review Board of the School of Public Health, Kinshasa University (ESP/CE/040/15; April 13, 2015) and the related longitudinal study investigating the impact of sanitation interventions was registered with the International Standard Randomised Controlled Trial registry (ISRCTN: 10419317). We obtained informed consent in a written form from the mothers/caretakers on behalf of the child under 4 years of age (U4C) enrolled in the study.

### Study setting.

**Figure 1.**
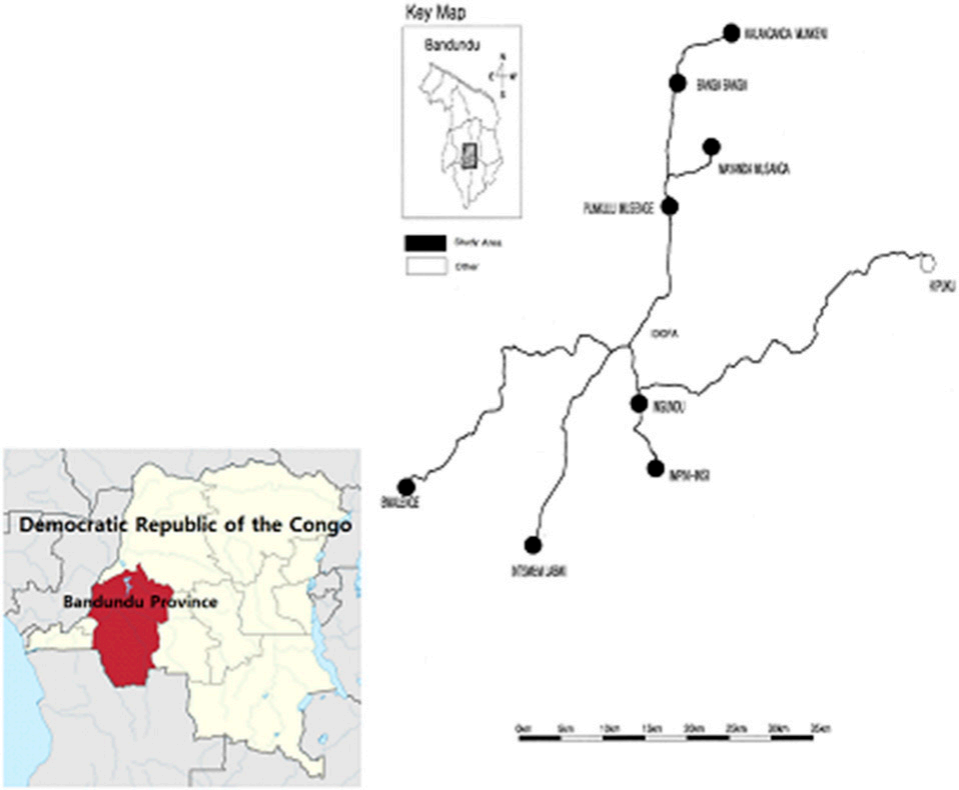
The study area in 18 quartiers in Idiofa, Bandundu, Democratic Republic of the Congo (• study area, ○ other). This figure appears in color at www.ajtmh.org.

The study was carried out in Idiofa Territory, Kwilu District, Bandundu State, the DRC, 655 km away from the capital city of Kinshasa (Figure 1). The territory has five cities and 12 sectors, with a population of 1.4 million. The Mubunda tribe is predominant, although the population is composed of diverse ethnic groups. Agriculture (maize, cassava, peanuts, and palm oil) is the main source of income, and people generally speak Kikongo.

The present study is part of a longitudinal study with a cluster randomized controlled trial investigating the effects of latrines along with a WASH project that will be conducted in Idiofa Territory, Bandundu Province, with the aim of reducing diarrhea among U5C by providing clean water and promoting household latrine improvement and relevant hygiene practices. A WASH committee was established in October 2014 for each target village. The WASH committee takes a role of community mobilization for latrine improvement and utilization, educating members of the community on hygienic practices and performing regular monitoring of sanitation coverage. The education for hygienic practices includes improvement and proper management of latrines, handwashing behaviors at four critical times (before eating, before cooking, after feeding child, and after defecating) and appropriate disposal of U5C feces. Based on the UNICEF Healthy Village Program in the DRC, we define improved sanitation in the project as latrines having 1) a pit with a depth of more than 1.5 m; 2) superstructure; 3) roof; 4) cement slab; 5) pit-hole cover; and 6) hand-washing facility. To encourage community-driven sanitation improvement, material subsidies for cement for making slabs and handwashing facilities will be provided only to the households who complete pit digging and construction of the superstructure and roof. This project incorporates a strong focus on hygiene practices because behavioral changes are necessary to ensure universal latrine usage.

### Sampling methods and sample size calculation.

#### Period prevalence of diarrhea.

Based on a preliminary survey, we estimated that the 7-day recall period prevalence of diarrhea in Idiofa Territory was 10%, and expected that our intervention will lead to a reduction of 2.5% (25% relative reduction). Although a systematic review^23^ showed 32% of relative reduction in diarrheal prevalence, we estimated the effect very conservatively. Assuming a coefficient of variation of 0.29, a 13% loss to follow-up, and a study power of 80%, our study was found to require 18 clusters (corresponding to 18 *quartiers*, i.e., subunits of communities) and total 720 children (360 children in each treatment and control arm of the study), using a standard formula.^24^ A two-stage cluster sampling method was used in this study. Among the 38 *quartiers* targeted by the WASH project in Idiofa Province, DRC, 18 were selected as a primary sampling unit. In these 18 *quartiers*, 720 households were sampled. The U4C in each household were registered for the longitudinal survey and given an identity number.

For this study, we are using the baseline sample from the longitudinal study with a cRCT design. We registered U4C to investigate the effects of latrines on the diarrheal reduction based on a 1-year follow-up plan of the longitudinal study with a cRCT study. We hypothesized that the households having a latrine would show significantly lower prevalence of child diarrhea compared with those not having a latrine. Given the sample size of 720 households, 20% diarrheal prevalence among households with latrine and 15% of diarrheal prevalence among households with no latrine produced a study power of 98%.

#### Household sample selection.

For the present study, 18 *quartiers* were randomly chosen from the 38 *quartiers* in which the Korea Environmental Corporation is implementing an integrated WASH project in collaboration with Water and Sanitation for Africa and the Service Nationale Hydraulique Rural (National Service for Rural Water Supply). A two-stage cluster sampling method was used for this study. Among the 38 project target *quartiers* for water pipe connection, sanitation, and hygiene intervention, 18 *quartiers* was selected as a primary sampling unit employing “Probability Proportional to Size.” Of the 18 *quartiers* residents, 720 households were sampled. The youngest U4C in each household was registered and assigned an identity number for the longitudinal study. The household-based baseline survey for the study was conducted in January 2015 in Idiofa Territory, Bandundu Province, DRC. Households with at least one U4C were eligible for this study. A total of 720 households were surveyed of the 1,399 households in the 18 *quartiers*, and 1,171 U4C were registered for the trial. No household refused to be registered in the study. For this study, we analyzed the data focusing only on the youngest child of each household surveyed.

We established three teams, each consisting of six data collectors and one supervisor. A *quartier* was divided into three blocks, and each block was assigned to a team. Households were selected based on convenience sampling. Each team member started enrolling households from the central location of the block until s/he reached her/his quota or the assigned boundary of the block.

### Study tool.

A household-based survey employing a structured questionnaire was conducted, and the enumerators made direct observations regarding the presence of a latrine, the type of the latrine, and the presence of human feces around the household compound. The questionnaire was developed in French and translated into Kikongo. Two lecturers with fluency both in French and Kikongo at Kinshasa University examined the questionnaire to validate it and to improve its accuracy. Although the survey was mainly conducted in Kikongo, we also used fluent speaker of Mubunda for a few groups of people who speak Mubunda to ensure we collect good quality data. Of the 18 enumerators, 10 had a bachelor’s degree corresponding to a 3-year course of study, three had a bachelor’s degree corresponding to a 5-year course of study, four had a secondary school diploma, and one was a doctor. The survey included information about the demographics and socioeconomic status of the head of the household and the caregiver, latrine ownership, the prevalence of diarrhea among the U4C and other household members, and the source of drinking water. The main outcome of interest was the 14-day reported prevalence of diarrhea in the youngest U4C of each household by parental report. We defined diarrhea when a child produced watery stools three or more times within 24 hours based on the UNICEF and WHO definition.^25^

Latrine coverage was assessed both by a question that asked whether the household had a latrine and direct observation. To assess latrine utilization, we separated household members by gender and age (under 4 years, from 4 to below 18 years, and 18 years or above). Based on the guideline of the Healthy Village Program (Village Assani) of the UNICEF DRC, we used an operational definition of an improved latrine for this study as follows: Improved latrines were those equipped with 1) a 1.5-m deep pit, 2) a cement slab, 3) a pit hole cover, 4) walls, 5) a roof, and 6) a handwashing facility. Several studies^26–28^ have demonstrated that latrine utilization was associated with its structure or quality. We believe that pit depth, a pit hole cover, a cement slab, and a handwashing facility would be expected to play a role in disrupting the transmission cycle. Latrines’ superstructures and roofs cannot play a direct role in preventing diarrheal transmission; however, we believe that the superstructure and roof are likely to facilitate latrine utilization of household members by providing privacy, especially for women and girls.

To explore the relationship between specific latrine improvements and diarrhea reduction, we also investigated the presence of human feces around the pit hole and examined the presence of flies and their quantity. If no flies were observed, the latrine was categorized as “no flies observed,” whereas if more than 10 flies were observed, we labeled it as “many flies were observed.” Unlike the operational definition of an improved latrine in the project implementation, we categorized the pit depth of latrines as less than 50 cm or more than 50 cm, because the baseline survey did not find any latrines with pits more than 1.5 m deep. In addition, no latrine was found to have a pit hole cover, a handwashing facility, or a cement slab. We thus categorized the latrines based on whether the pit depth was more than 50 cm, the presence of feces around the pit hole, the presence of flies, the presence of a superstructure, and the presence of a roof. For measuring handwashing behavior, we used the combined methods of self-report and rapid household observation. The method of self-report may exaggerate the true behavior of handwashing because it may be perceived as socially desirable behavior. During the household survey, we conducted rapid household observations on the availability of soap and water and the presence of these tools at dedicated handwashing stations. When the soap is not in place, we asked the respondent to bring it to the interviewer and checked whether less than 1 minute was required. For self-report in the household survey, we used unprompted measures to check self-reported handwashing behavior with soap at four critical times by asking “under which circumstances did you wash your hands with soap?”

### Data analysis.

We sought to identify demographic and socioeconomic characteristics (tribe, religion, occupation, education level, number of U4C, and average age of U4C) of study population as well as the risk factors and behavioral factors associated with the prevalence of diarrhea (drinking water quality, household hygiene, and environmental hygiene). Multivariable logistic regression models were used to examine the association between the presence of a household latrine and the prevalence of diarrhea in U4C. We conducted the analysis by comparing the prevalence of diarrhea in households with and without a latrine, and also comparing the prevalence of diarrhea according to each latrine component. We did not adjust for household socioeconomic status when investigating the effects of latrine ownership on child diarrheal prevalence. According to Sima and others’ risk category modeling,^29^ household SES variables are mediators, affecting household hygiene. In other words, per-capita income below poverty was the common predictor for decreased household hygiene, which means the poverty-diarrhea prevalence link is mediated by household hygiene. Therefore, household-level SES is correlated with the water source, sanitation facilities, and the handwashing behaviors of caregivers. We thus perceive that the water source, sanitation facility, and handwashing behaviors are on a pathway between household level SES and child diarrheal infection. Therefore, household SES may not be a confounder between latrine ownership and child diarrhea prevalence. Data analyses were conducted using SAS 9.2 (SAS Institute Inc, Cary, NC).

## RESULTS

### Sampled population.

Table 1 provides information on the 720 households from 18 *quartiers* that were included in the study, representing 51.47% of all households in the study area. Among the 720 households enrolled in the study, 461 (64.00%) had a latrine, and the remaining 259 (36.00%) did not.

**Table 1:** General characteristics of households of the study population

			Household with latrine		Household without latrine		
Household(H/H) characteristics			Number or mean	% or SD	Number or mean	% or SD
Demographic and socioeconomic status		Total number	461	100.00	259	100.00
	H/H head’s gender	Male	419	90.90	223	86.10
	HH head’s age	Years	41.63	11.52	38.29	10.55
	H/H head’s tribe	Mubunda	453	98.30	255	98.50
	H/H head’s religion	Christian	435	94.40	247	95.40
	H/H head’s main occupation	Farmers	216	46.90	128	49.40
	Caregiver’s gender	Female	461	100.00	259	100.00
	Caregiver’s age	Years	31.43	9.09	29.18	8.56
	Caregiver’s education level	Primary completed	293	63.60	155	59.80
	Caregiver’s link with head	Wife	393	85.20	223	86.10
	No of U4C		1.67	0.78	1.56	0.63
	Gender of youngest U4C	Male	252	54.70	123	47.50
	Age of youngest U4C	Months	20.06	14.36	19.58	13.42
	Number of H/H members		6.4	2.34	5.64	2.05
	H/H income per month	US$	26.81	53.67	16.86	22.04
Water related factors	Water source for drinking	Protected water	16	3.50	7	2.70
	Water quantity for fetching per day per household	(L)	58.3	50.66	55.7	56.56
	Cleaning water container	Yes	434	98.20	243	96.00
	Frequency of cleaning water container	Daily	257	59.20	138	56.80
		Every other day	106	24.40	64	26.30
	Water treatment	Yes	24	5.20	7	2.70
	Average time for fetching drinking water	Minutes	103.47	14.94	107.72	14.78
Handwashing practices	Handwashing practices	Before eating	435	94.40	244	94.20
		After defecating	358	77.70	278	68.70
		Before cooking	168	36.40	71	27.40
		Before feeding a child	24	5.20	7	2.70
		After cleaning a child’s buttocks	5	1.01	4	1.54
	Handwashing with water and soap		417	90.50	221	85.30

H/H = household; SD = standard deviation.

### Basic characteristics of study population.

Most of the heads of household in the project area were from the Mubunda tribe and were Christian, and almost half of them were farmers. The average age of the youngest U4C in the 720 households was approximately 20 months. Only a minority of households had access to safe water sources (protected springs, boreholes, or rainwater), and the majority of the people drank water from rivers or unprotected streams. They reported that they spent more than 100 minutes for fetching water on average. In contrast with high percentage of handwashing practice before eating, only a paucity of people reported they wash their hands before feeding a child or after cleaning a child’s buttocks.

### Latrine coverage and characteristics.

None of the latrines in the 461 households with latrines satisfied the definition of an improved latrine according to the guidelines of the Healthy Village Program (Village Assani) of the UNICEF DRC, which mandate that an improved latrine should be equipped with a handwashing facility, a cement slab, a pit hole cover, a superstructure, and a roof, and have a pit depth of at least 1.5 m. On the village level, latrine coverage ranged from 51.9% to 80.4% and on the *quartier* level, from 40.7% to 88.2%.

As described in the Methods section, since no latrines were found to have a pit hole cover, handwashing facility, or cement slab, we categorized the latrines based on whether they had a pit depth greater than 50 cm, the presence of feces around the pit hole, the presence of flies, the presence of a superstructure, and the presence of a roof. We also calculated the percentage of households with a latrine according to each element. First, we compared the prevalence of diarrhea in U4C between households with and without a latrine. Second, we compared the prevalence of diarrhea in U4C in households with a latrine, according to different types of latrines.

### Comparison between households with and without latrines; comparison within households having a latrine with and without specific latrine components.

Association between latrine ownership and the prevalence of diarrhea U4C in households with access to a latrine was less likely to contract diarrhea than their counterparts living in households without a latrine, regardless of the type of latrine installation (Table 3). The odds of U4C contracting diarrhea were 30% lower in households with a latrine than in households without a latrine (crude odds ratio [OR] = 0.67, 95% confidence interval [CI] = 0.49–0.91; adjusted OR = 0.70, 95% CI = 0.50–0.99).

Flies have been shown to be an important transmission vector in the transmission pathway between human feces and diarrhea. In households where flies were not observed inside the latrine, children were less likely to have diarrhea than in households with a latrine where flies were present or in households without a latrine (crude OR = 0.63, 95% CI = 0.42–0.95; adjusted OR = 0.64, CI = 0.43–0.96). This association was also significant when households with fly-free latrines were compared with households with latrines in which flies were observed (crude OR = 0.63, 95% CI = 0.42–0.95; adjusted OR = 0.51, 95% CI = 0.32–0.82).

**Figure 2.**
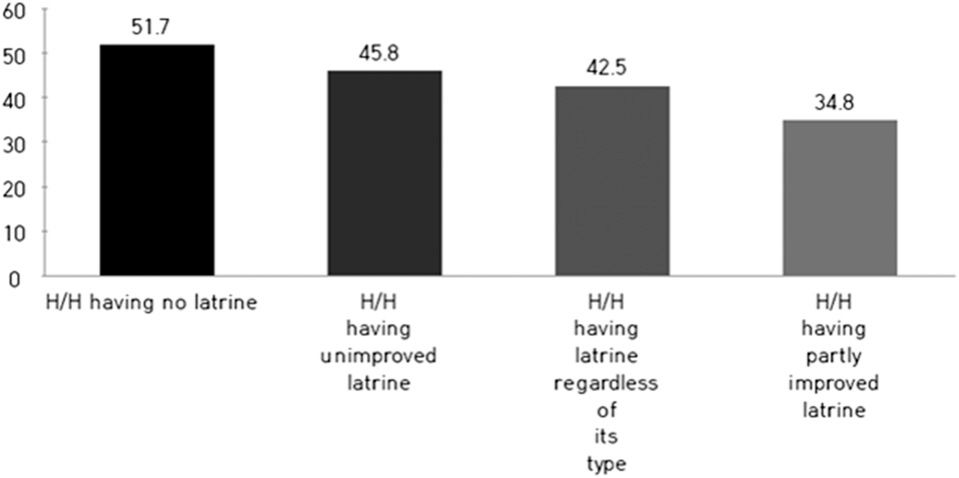
Child diarrheal prevalence by the improved status of latrine (x axis, latrine type; y axis, diarrheal prevalence [%]).

**Figure 3.**
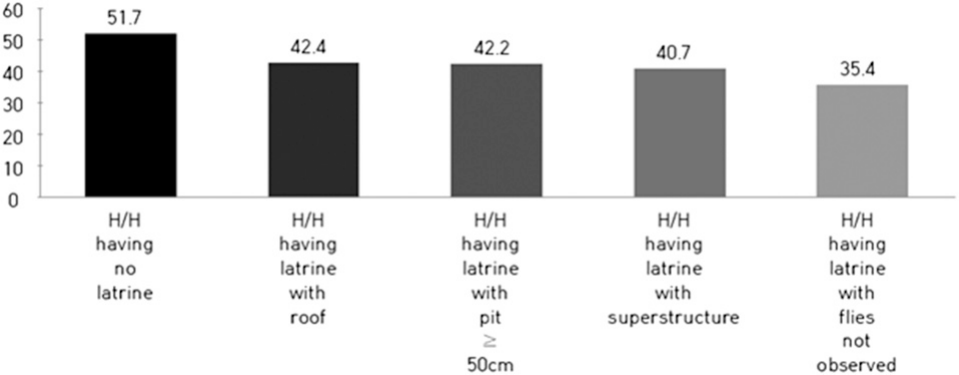
Child diarrheal prevalence by latrine component or management status (x axis, latrine type; y axis, diarrheal prevalence [%]).

The analysis shows that if a latrine were to be better equipped or more appropriately managed, it could be more effective in preventing child diarrhea (Table 2 and Figures 2 and 3). Households who have a latrine with a superstructure, roof, and no flies (we defined this as “a partly improved latrine”) were found to be 52% less likely to report cases of diarrhea than households with unimproved latrines (crude OR = 0.63, 95% CI = 0.42–0.95; adjusted OR = 0.48, 95% CI = 0.31–0.76), which are all the other latrines not included in the partly improved latrine category (Table 4). We have observed the profound protective effect of latrines with a superstructure. Intuitively, a latrine superstructure seems less likely to play a role than other components in disrupting the cycle of oral–fecal transmission. The implication of a latrine superstructure is described in the Discussion section.

**Table 2:** Association between latrine ownership and diarrheal prevalence (comparison between households with and without latrines)

		Diarrhea prevalence
	% (*N*)	
H/H having no latrine	36.0 (259)	51.7
H/H having latrine	64.0 (461)	42.5
H/H having no latrine or H/H having latrine with no superstructure	40.0 (288)	53.5
H/H having latrine but with no superstructure	6.29 (29)	69.0
H/H having latrine with superstructure	60.0 (432)	40.7
H/H having no latrine or H/H having latrine with no roof	46.2 (333)	49.8
H/H having latrine but with no roof	16.1 (74)	43.2
H/H having latrine with roof	53.8 (387)	42.4
H/H having no latrine or H/H having latrine with pit < 50 cm	50.0 (360)	49.4
H/H having latrine but with pit < 50 cm	21.9 (101)	43.6
H/H having latrine with pit ≥ 50 cm	50.0 (360)	42.2
H/H having no latrine or H/H having latrine with feces observed	49.0 (353)	47.9
H/H having latrine but with feces observed	20.4 (94)	37.2
H/H having latrine with feces not observed	51.0 (367)	43.9
H/H having no latrine or H/H having latrine with flies observed	77.2 (556)	48.9
H/H having latrine but with flies observed	64.4 (297)	46.5
H/H having latrine with flies not observed	22.8 (164)	35.4
H/H having no latrine or H/H having unimproved latrine	80.8 (582)	48.5
H/H having unimproved latrine	70.1 (323)	45.8
H/H having partly improved latrine^1^	19.2 (138)	34.8

A partly improved latrine is defined as a latrine with a superstructure, roof, and no flies observed, and all the other latrines not partly improved latrines are unimproved latrine in this study.

**Table 3:** Association between latrine ownership and diarrheal prevalence

			Crude			Adjusted^1^		
		Reference	OR	95% CI		OR	95% CI	
Latrine structure	H/H having latrine	H/H having no latrine	0.670	0.493	0.91	0.701	0.496	0.992
	H/H having latrine with superstructure	H/H having no latrine or H/H having latrine with no superstructure	0.578	0.428	0.781	0.625	0.444	0.880
	H/H having latrine with roof	H/H having no latrine or H/H having latrine with no roof	0.671	0.434	1.036	0.800	0.572	1.120
	H/H having no latrine or H/H having latrine with pit ≥ 50 cm	H/H having latrine with pit < 50 cm	0.919	0.564	1.5	0.860	0.617	1.199
Latrine maintenance	H/H having latrine with feces not observed	H/H having no latrine or H/H having latrine with feces observed	1.08	0.669	1.744	0.856	0.613	1.195
	H/H having latrine with flies not observed	H/H having no latrine or H/H having latrine with flies observed	0.629	0.417	0.949	0.637	0.425	0.955
	H/H having partly improved latrine	H/H having no latrine or H/H having unimproved latrine	0.624	0.416	0.936	0.617	0.402	0.947

CI = confidence interval; OR = odds ratio.

Adjusted for the accessibility of protected water and handwashing behavior at critical times (before eating, before cooking, and after defecating); child’s age and sex; household monthly income, and caregiver’s education level.

**Table 4:** Association between latrine ownership and diarrheal prevalence (comparison among households having a latrine with and without specific latrine components or latrine management)

			Crude			Adjusted		
		Reference	OR	95% CI		OR	95% CI	
Latrine Structure	H/H having latrine with superstructure	H/H having latrine but with no superstructure	0.295	0.131	0.663	0.348	0.138	0.880
	H/H having latrine with roof	H/H having latrine but with no roof	0.671	0.434	1.036	0.907	0.491	1.676
	H/H having latrine pit ≥ 50 cm	H/H having latrine but with pit < 50 cm	0.919	0.564	1.5	0.838	0.500	1.403
Latrine Maintenance	H/H having latrine feces not observed	H/H having latrine but with feces observed	1.08	0.669	1.744	1.090	0.628	1.891
	H/H having latrine fly not observed	H/H having latrine but with fly observed	0.629	0.417	0.949	0.513	0.320	0.821
	H/H having partly improved latrine	H/H having unimproved latrine	0.631	0.417	0.953	0.484	0.307	0.763

CI = confidence interval; OR = odds ratio.

Adjusted for the accessibility of protected water and handwashing behavior at critical times (before eating, before cooking, and after defecating); child’s age and sex; household monthly income, and caregiver’s education level.

We believe that the lack of an effect of latrines with no feces around the pit hole arose from a measurement error. For the baseline survey, we were able to carry out direct observation only once. The presence of feces around the pit hole could vary greatly depending on the duration after cleaning by an individual household. In contrast, the presence or absence of flies could be expected to be flat over a certain period. With only one direct observation, we were not able to properly represent the presence or absence of feces around the pit hole. We recommend repeated observations to measure the cleanliness inside a latrine.

### Characteristics of latrine utilization.

The majority of households (98.3%) with a latrine reported using their latrine, although this was generally not the case for all household members. Only 20 households (4.3%) reported that their latrine was used by all household members. The overwhelming majority (433, 95.7%) of households with a latrine reported that their U4C did not use the latrine; of these children, 84.5% defecated around the corner of the house or around the latrine, whereas others (11.3%) defecated in diapers. Most of the latrines (407) had been constructed less than 3 years previously. The respondents from 230 households without a latrine (89.2%) reported defecating in their neighbors’ latrine, and 29 (10.8%) reported defecating in open areas, such as the river, forest, or bush.

The respondents from 214 households without a latrine (82.6%) stated that they were willing to construct a latrine within the next 12 months, whereas the respondents from 239 households with a latrine (51.8%) stated that they planned new latrine construction. Among the respondents without a latrine, the main reasons for being interested in building a latrine were as follows: to have their own latrine, avoiding “shame,” and avoiding infection. The respondents from approximately half of the households with a latrine expressed an interest in future construction because their latrine pit was full or frequently collapsed. Among the households without a latrine, respondents who had no plans for future construction reported the main barrier to constructing a latrine was lack of funds.

## DISCUSSION

This study demonstrates that even suboptimal latrines are associated with significant improvements in health-related parameters. In the study area, no household had an improved latrine that fully met all of the criteria established by UNICEF DRC (a superstructure, a roof, a cement slab, a pit hole deeper than 1.5 m, a pit hole cover, and a handwashing facility within 1 m). However, the households with a latrine showed a lower prevalence of diarrhea among U4C than households without a latrine, regardless of the latrine type. This suggests that the latrine itself provides health benefits, even if it is not fully equipped with the elements necessary to be considered improved. This cannot be directly interpreted as the effect of using a latrine compared with open defecation, because the majority of households without a latrine reported utilization of a neighbor’s facility instead of open defecation, although we cannot rule out the possibility of courtesy bias. This result adds to the body of evidence demonstrating the need for encouraging the uptake of latrines at the household level.

Our study found that partially improved latrines were clearly associated with a lower diarrheal prevalence. The prevalence of diarrhea in households that had a latrine with superstructure and a roof, and with no flies, was lower, when compared with the prevalence in households having a latrine without some or all of these three components. The results reinforce the importance of particular latrine components and latrine management in preventing diarrheal disease among U5C. These results reinforce the importance of each element required for an improved latrine in prevention of diarrheal disease among U5C.

We also explored the relationship of the presence of feces and flies around the pit hole with the prevalence of diarrhea. We found that the presence of flies was strongly associated with a higher prevalence of diarrhea. This association was observed both in a comparison among households with a latrine and in a comparison among all households.

The majority of households that did not have a latrine identified the lack of funds as the main factor for not building a latrine (data not shown). In addition, the members of households without a latrine spent more time fetching drinking water. This may have been caused by a lack of transportation in the lower-income group, but we did not explore the means of transportation for obtaining water. Unlike previous studies, this study did not find latrine ownership to be associated with the education level of the head of household or the caregivers. Appropriate handwashing practices were more frequently reported among households with a latrine. Because of the cross-sectional design of this study, we could not infer whether low-income status could cause a lower rate of handwashing, or how the low prevalence of handwashing could be related to the relative absence of latrines in this group. Nonetheless, we suggest that the low rate of both handwashing and latrine ownership could be associated with a lack of awareness of hygiene and sanitation in this group.

Respondents from the majority of households with no latrine reported that they used a neighbor’s facility. However, it is possible that they exaggerated their latrine use, which is especially likely since many respondents stated that “to avoid being ridiculous” or “to have their own latrine” were their main reasons for planning to construct their own latrine in the next 12 months. Additionally, it can be inferred that social norms and/or a shame culture played a role in motivating some community members to adopt household latrines.^30^

Very few latrines were used by all household members, and almost all U4C did not use the household latrine. The majority of young children were reported to defecate around the corner of their house, regardless whether they were ambulatory or non-ambulatory. Open defecation was practiced by U4C more often than by other age groups. This practice may be a major risk factor for exposing household members to pathogens. Our findings demonstrate that a pressing need exists for developing child-friendly latrines for ambulatory children and encouraging the safe disposal of children’s feces.

We used a 14-day recall period for the cross-sectional study, which might have increased the subjectivity of reporting. However, there must be less plausibility of a significant difference in recall bias when measuring reported diarrheal prevalence between the two comparison groups for this study. The cross-sectional design of the study does not allow causal inferences to be drawn between latrine coverage and the prevalence of diarrhea. The cohort members, comprising the youngest U4C in 720 households, will undergo follow-up to explore the effects of increased latrine coverage on the prevalence of diarrhea in children. We expect that our future study will present evidence quantifying the effects of latrines on health outcomes. To assess the association between latrine coverage and the prevalence of diarrhea, we adjusted for the type of water source (protected or unprotected) and handwashing practices at three critical times (before eating, after defecation, and before cooking). It is possible that we did not adjust for some confounders. A 7-day recall period or a shorter recall period has been recommended in diarrhea trials. Using a 14-day recall period to assess the prevalence of diarrhea may have introduced recall bias, which is another limitation of this study.^31^ However, there must be less plausibility of significant difference in recalling bias when measuring reported diarrheal prevalence between the two comparison groups for this study. The next stage of our cluster-randomized trial will involve adopting a sanitation calendar that caregivers can use for daily self-recording, which we expect to overcome the recall bias that may accompany the use of 7-day or 14-day reported diarrhea as a metric.

For latrine utilization, we used combined methods. First, we checked whether there were footprints and/or grass on the path from the house to a latrine. Second, we checked whether spider webs existed at the entrance of the latrine. We also checked whether the feces were very dry or not, and we checked for an odor. We categorized a household into the group utilizing latrines only if there was no discrepancy in all these various measurement results, including those of the survey. There might have been interobserver bias since we cannot rule out the possibility that the observation results could have varied depending on the data collector’s subjective observation. There is more a robust way of measuring handwashing behavior such as bars of soap with motion sensors, microbiological measures of handwashing. We measured handwashing behavior to adjust its confounding effect between latrine ownership and diarrheal prevalence. There must be less plausibility of a significant difference in measurement of handwashing behavior between the two comparison groups for this study. Unfortunately, we could not analyze the effect of “open defecation free,” because not a community had achieved open defecation free status at the community level.

This study adds valuable information to the limited evidence that exists regarding the effects of latrines on health parameters by demonstrating that latrines have health benefits regardless of whether they are improved, as well as by elucidating the most essential components of improved latrines. So far, although the public health importance of sanitation has been repeatedly raised, evidence has not been rigorously collected concerning the essential elements with which an improved latrine should be equipped. The study findings could stimulate more robust studies investigating what components comprise an “improved latrine.” Future cluster-randomized trials will use a more rigorous methodology to permit causal inferences to be drawn.
